# Improving the Power Conversion Efficiency of Carbon Quantum Dot-Sensitized Solar Cells by Growing the Dots on a TiO_2_ Photoanode In Situ

**DOI:** 10.3390/nano7060130

**Published:** 2017-05-31

**Authors:** Quanxin Zhang, Geping Zhang, Xiaofeng Sun, Keyang Yin, Hongguang Li

**Affiliations:** 1 State Key Laboratory of Solid Lubrication & Laboratory of Clean Energy Chemistry and Materials, Lanzhou Institute of Chemical Physics, Chinese Academy of Sciences, Lanzhou 730000, China; qxzhang@licp.cas.cn (Q.Z.); sunxiaofengvip@licp.cas.cn (X.S.); yinky110403@163.com (K.Y.); 2 Key Laboratory of Colloid and Interface Chemistry & Key Laboratory of Special Aggregated Materials, Shandong University, Ministry of Education, Jinan 250100, China; zgp0609@mail.sdu.edu.cn

**Keywords:** carbon quantum dot, solar cell, sensitized, hydrothermal, power conversion efficiency

## Abstract

Dye-sensitized solar cells (DSSCs) are highly promising since they can potentially solve global energy issues. The development of new photosensitizers is the key to fully realizing perspectives proposed to DSSCs. Being cheap and nontoxic, carbon quantum dots (CQDs) have emerged as attractive candidates for this purpose. However, current methodologies to build up CQD-sensitized solar cells (CQDSCs) result in an imperfect apparatus with extremely low power conversion efficiencies (PCEs). Herein, we present a simple strategy of growing carbon quantum dots (CQDs) onto TiO_2_ surfaces in situ. The CQDs/TiO_2_ hybridized photoanode was then used to construct solar cell with an improved PCE of 0.87%, which is higher than all of the reported CQDSCs adopting the simple post-adsorption method. This result indicates that an in situ growing strategy has great advantages in terms of optimizing the performance of CQDSCs. In addition, we have also found that the mechanisms dominating the performance of CQDSCs are different from those behind the solar cells using inorganic semiconductor quantum dots (ISQDs) as the photosensitizers, which re-confirms the conclusion that the characteristics of CQDs differ from those of ISQDs.

## 1. Introduction

The worldwide energy crisis evokes an unprecedented enthusiasm for the development of an apparatus for efficient light-to-electricity conversion. Dye-sensitized solar cells (DSSCs), together with organic photovoltaics (OPVs), are generally considered two types of third-generation energy-harvesting devices. There is an enthusiasm for engineering the electrolytes, i.e., from non-aqueous systems to aqueous ones [1,2,3,4]. As another key component in DSSCs, the photosensitizer is responsible for light absorption and the subsequent electron-transfer reactions, and its quality thus determines the power conversion efficiency (PCE). Inorganic semiconductor quantum dots (ISQDs), such as CdS, CdSe, and PbS, represent an important class of photosensitizers. Compared to DSSCs employing Ru–polypyridine complexes or low-molecular-weight organic dyes as photosensitizers, ISQD-sensitized solar cells (ISQDSCs) are expected to have low costs and relatively high PCE [5,6]. A big disadvantage of ISQDSCs, however, is their intrinsic toxicity caused by the presence of heavy metal elements in ISQDs. Developing new types of quantum dots that are environmentally benign is necessary for further realizing the potential of this new branch of DSSCs.

In recent years, carbon quantum dots (CQDs), which are photoluminescent (PL) carbon nanomaterials that have spherical shapes and sizes below 10 nm, have received extensive attention due to their easy preparation and tunable PL properties [7]. More importantly, CQDs are nontoxic for both environment and biological systems, which is advantageous over ISQDs. Based on these virtues, CQDs exhibit great potential applications in various fields and have been used in bioimaging [8], ion detection [9,10], PL inks [11,12,13], and coatings for light-emitting diodes (LEDs) [14]. Attempts to integrate CQDs in photovoltaics have also been made [15,16,17]. Specifically in DSSCs, CQDs have been used as co-sensitizers together with ISQDs [18] or organic dyes [19], and the CQD-based solar cells were found to show superior performances.

Applying CQDs as the sole photosensitizer in DSSCs, compared to using CQDs as an additive in mixed photosensitizers, is more interesting but also more challenging. In a seminal work, Mirtchev et al. integrated TiO_2_ photoanode with CQDs obtained from acid dehydration of γ-butyrolactone, and a PCE of 0.13% was obtained [20]. Zhang et al. also reported the preparation of DSSCs using N-doped CQDs as photosensitizers, and the PCE was found to be the same as that reported by Mirtchev et al. (i.e., 0.13%) [21]. Briscoe et al. used three different types of biomass-derived CQDs as photosensitizers, which were hybridized with ZnO as photoanodes. However, the highest PCE of the solar cells is even lower (0.077%) [22]. Recently, Margraf et al. reported CQD-based DSSCs with a PCE of 0.24% [23]. They found that the pH value of the aqueous solution where CQDs and TiO_2_ photoanodes were integrated plays an important role in governing the performance of the solar cell, while extending the light absorption of CQDs towards longer wavelengths does not help. Wang et al. also prepared CQD-based DSSCs by integrating CQDs and TiO_2_ photoanodes in acetone. A PCE of 0.79% was obtained [24]. There are also research interests on edge-bound nanometer-size graphene pieces with lateral dimensions less than 100 nm termed graphene quantum dots (GQDs), which can be viewed as close relatives of CQDs. In this case, two works using GODs as photosensitizers in DSSCs have been presented. Yan et al. synthesized GQDs from small organic molecules, and integrated them with TiO_2_ photoanodes in a toluene/ethanol mixture [25]. The solar cell exhibits a considerable open-circuit voltage (*V*_OC_, 0.48 V) and fill factor (*FF*, 0.58). However, it suffers from low short-circuit current density (*J*_SC_, 0.2 mA/cm^−2^), which gives a final PCE of ~0.06%. Ji et al. prepared GQDs on TiO_2_ via a Scholl reaction from a preadsorbed polyphenylene precursor functionalized with surface anchoring groups, and a PCE of 0.87% was obtained [26], which is slightly higher compared to that reported for the solar cells sensitized by CQDs.

It is clear that research on CQD-sensitized solar cells (CQDSCs) is still in its infancy. Although great efforts have been devoted as mentioned above, further improvement of the PCE is necessary. More importantly, the use of acetone in the preparation of the solar cell with the highest PCE reported to date (0.79%) [24] is not environmentally benign and is incompatible with the majority of the CQDs, which are water-soluble. On the other hand, the GQD-sensitized solar cells, though exhibiting a slightly higher PCE (0.87%) [26] than that of CQDSCs, suffer from multi-step, time-consuming synthesis of the photosensitizers, which will significantly raise the costs of resulting DSSCs.

One big challenge causing the unsatisfactory PCE of CQDSCs is the low affinity between TiO_2_ (or ZnO) and CQDs. In previous studies, the CQDs were always synthesized first, followed by integration with the photoanode by post-adsorption [20,22,23,24,25,26] or hydrothermal treatment [21] in bulk solutions. This simple treatment leads to a poor adsorption of the CQDs onto the TiO_2_-coated photoanode. Herein, we provide a new engineering method for a CQD-based photoanode, where CQDs were grown in situ onto the TiO_2_ surface. We will show that, by using this strategy, the surface coverage of TiO_2_ by CQDs can be significantly improved, leading to an impressive performance of the solar cell.

## 2. Results and Discussion

**Figure 1:**
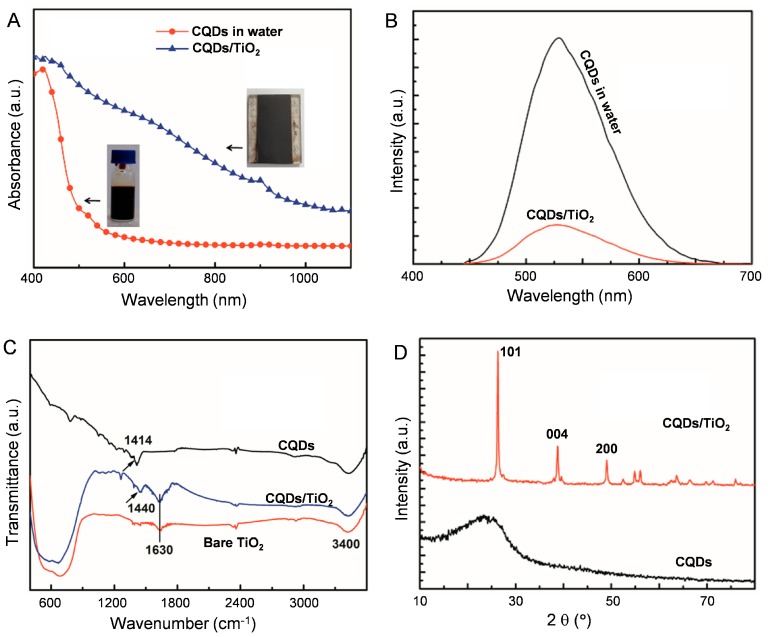
(**A**) UV-vis-NIR absorption of as-prepared carbon quantum dots (CQDs) in water and CQDs/TiO_2_ on fluorine-doped tin oxide (FTO) glass. Insets are photos of the CQDs aqueous solution and CQDs/TiO_2_-coated photoanode; (**B**) PL spectra of CQDs in water and CQDs/TiO_2_ on FTO glass, excited at 440 nm; (**C**) FTIR spectra of solid CQDs, TiO_2_- and CQDs/TiO_2_-coated FTO glass; (**D**) XRD pattern of solid CQDs and CQDs/TiO_2_-coated FTO glass.

For the in situ growth of CQDs, the TiO_2_-coated fluorine-doped tin oxide (FTO) glass was vertically immersed in a 10 mL aqueous solution containing citric acid (CA, 0.5 mol·L^−1^) and ethanediamine (EDA, 0.5 mol·L^−1^). The system was then sealed in an autoclave and pyrolized at 180 °C for 24 h. After that, the as-prepared CQDs/TiO_2_ was rinsed with deionized water thoroughly and dried in air. Here, CA and EDA were selected as the precursors for CQD production because the synthetic procedures of CQDs from this combination have been well-established [13]. The CQDs/TiO_2_ hybridized photoanode (abbreviated to CQDs/TiO_2_ hereafter) appears as a dark-brown film (inset of Figure 1A), which is in sharp contrast to the control experiment where the TiO_2_ photoanodes were found to be only slightly colored if physical adsorption was applied. This indicates that, by adopting the in situ growth method, the amount of CQDs anchored on the photoanode has been significantly improved. From the UV-vis-NIR absorption spectra, it can be seen that CQDs/TiO_2_ exhibits extensive optical absorption throughout the visible and NIR regions, while the absorptions of CQDs alone are mainly located in the UV region (Figure 1A). PL spectra indicate that, after being integrated onto the TiO_2_ photoanode, the fluorescence of CQDs is quenched (Figure 1B). The shift of the absorption towards longer wavelengths and the fluorescence quenching of CQDs on the photoanode indicate a more compact arrangement of the dots in the film state. The lower emission intensity of CQDs on the photoanode compared to those in the bulk solution is also indicative of a lower recombination rate of the excitons, which is preferable when using the materials as photosensitizers [27]. 

The changes in the optical properties of CQDs after being integrated with TiO_2_ might also be caused by the interactions between them. To test this possibility, FTIR measurements were carried out, as shown in Figure 1C. The peak at 1414 cm^−1^ assigned to the C–O stretching vibration for CQDs shifts to 1440 cm^−1^ in CQDs/TiO_2_, which could be caused by the interaction between CQDs and TiO_2_ [28]. Peaks at 1630 cm^−1^ and 3400 cm^−1^, which appear in both bare TiO_2_ and CQDs/TiO_2_, could be caused by vibrations of surface adsorbed water. From X-ray diffraction (XRD) measurements shown in Figure 1D, mainly peaks related to anatase TiO_2_ can be detected (2*θ*: 25.1°, 38.1°, 47.9°) in CQDs/TiO_2_. CQDs alone only show a broad band centered around 25°, which indicates the low crystallinity of the synthesized CQDs.

**Figure 2:**
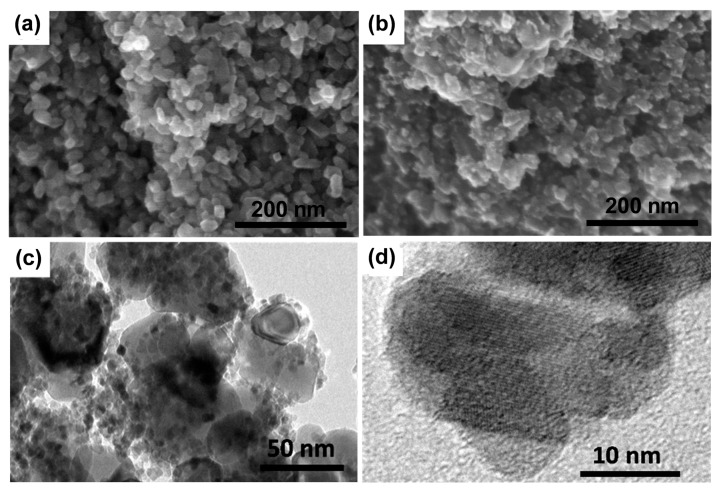
(**a**,**b**) SEM images of TiO_2_-coated photoanode before and after in situ growth of CQDs, respectively. (**c**,**d**) TEM images in different magnifications of the CQDs/TiO_2_ photoanode.

The morphologies of the TiO_2_-coated photoanode before and after CQDs hybridization have been investigated by scanning electron microscopy (SEM) observations. The TiO_2_ nanoparticles organized on FTO glass into a porous structure (Figure 2a). When CQDs were integrated with the TiO_2_-coated photoanode, the presence of CQDs can be confirmed (Figure 2b). Importantly, the TiO_2_ film remains porous after it is hybridized with CQDs without obvious pore blocking, which will facilitate the penetration of the electrolyte in the solar cell. To get further details, the CQD-sensitized photoanode was further checked by transmission electron microscopy (TEM) observations (Figure 2c,d). It can be seen that the CQDs grown in situ on TiO_2_ surfaces have sizes of 2–6 nm, and most of them distribute evenly. The lack of severe aggregation highlights the advantage of the in situ growth method.

**Figure 3:**
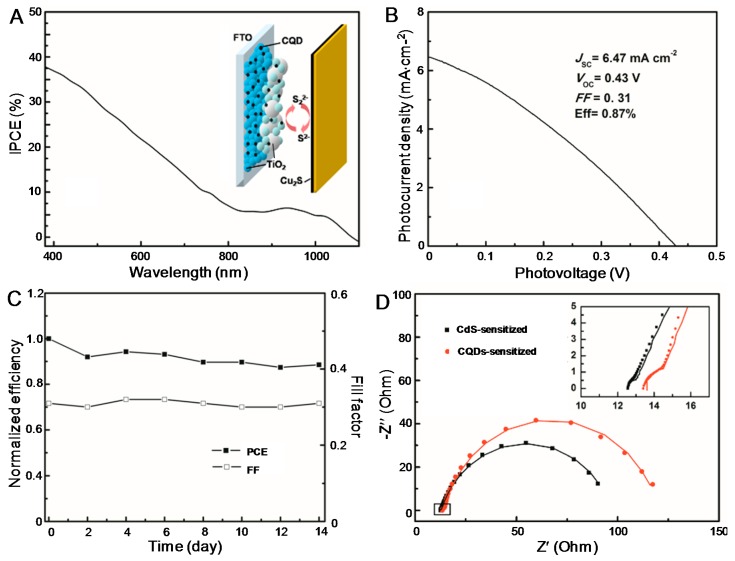
(**A**) IPCE spectra and (**B**) *JV* curve of the CQDSC. Inset of (**A**) shows the structure of the solar cell. (**C**) Variation of the *FF* and PCE of the CQDSC as a function of storage time. (**D**) EIS results of the CdS-sensitized solar cell and CQDSC. The symbols are experimental results, while the solid lines are fitted curves based on the equivalent circuit developed previously [26].

To construct the solar cell, Cu_2_S on brass was selected as the counter electrode and polysulfide consisting of 1 mol·L^−1^ Na_2_S and 1 mol·L^−1^ S in aqueous solution was used as the electrolyte. The selection of this cell structure is in favor of comparison with previously reported CdS-sensitized solar cells [29] wherever necessary. An illustration of the cell structure is given in the inset of Figure 3A. The IPCE spectrum shown in Figure 3A matches the absorption spectrum of CQDs/TiO_2_, indicating that the photocurrent was generated via the photoexcitation of CQDs. From the *JV* curve, a *J*_SC_ of 6.47 mA·cm^−2^, a *V*_OC_ of 0.43 V, and an *FF* of 0.31 were obtained, which leads to a PCE of 0.87% (Figure 3B). To the best of our knowledge, this PCE is the highest reported efficiency for solar cells sensitized by both CQDs and GQDs (Table S1), which is mainly attributed to the impressively high *J*_SC_. As the CQDs were grown in situ, the high *J*_SC_ could benefit from the improved contact between the CQDs and TiO_2_. Investigations on parallel-prepared cells indicated that *J*_SC_, *V*_OC_, and PCE of the cells are reproducible with variations less than 8%. To test the long-term stability of the solar cell, it was stored at room temperature in the dark for up to 14 days. No significant decrease of both PCE and *FF* could be observed (Figure 3C), revealing the good stability of the cell.

We noticed that the *FF* of our solar cell is lower than that of previously reported cells (Table S1). As researchers tend to use different types of CQDs and the structure of the solar cells also varies, a strict comparison between different CQDSCs reported to date could be difficult. Previously, using the same cell structure, we have constructed a CdS-sensitized solar cell [29], which is selected for comparison with the CQDSC reported in this work for further insight. The *J*_SC_, *V*_OC_, *FF*, and PCE of the CdS-sensitized solar cell have been determined to be 6.57 mA·cm^−2^, 0.435 V, 0.53, and 1.52%, respectively. Again, it is found that the *FF* of the CQDSC is lower. It is known that the performance of the solar cell is heavily influenced by its internal charge transfer. To find out the reason for the relatively low *FF* of the CQDSC, electrochemical impedance spectroscopy (EIS) measurements on the two cells sensitized by CQDs and CdS were carried out. In Nyquist plots, a dominant semicircle was observed in the mid and low frequencies together with a small one at high frequencies. We temporarily adopted the equivalent circuit developed previously [29] to fit the data using Zview software, and fairly good fittings are obtained (the solid lines). It is known that the big semicircle reflects the charge transfer resistance and interfacial capacitance at the counter electrode/electrolyte interface, while the small one represents the charge recombination resistance and constant phase element at the photoanode/electrolyte interface. Compared to the CdS-sensitized solar cell, the CQD-sensitized cell suffers from a larger charge transfer resistance as evidenced from the much larger semicircle in the mid and low frequencies, which might account for its lower *FF* and PCE. This conclusion is consistent with the remarks given in Margraf’s work, where the authors claimed that the balance between the charge recombination and regeneration processes is not ideal in CQDSCls [23].

In ISQDSCs, it has been demonstrated that surface passivation of the photoanode by ZnS has a positive effect on the performance of the solar cells [30]. When this method was applied to the CQDSCs presented in this work, however, decreased *J*_SC_ and *V*_OC_ values were observed, which results in a lower PCE of 0.64% (Figure S1). The effects of photoanode annealing on the performance of the CQDSCs were also examined. Briefly, the CQDs-hybridized photoanode was heated in a muffle furnace under nitrogen for 10 min at 350 °C and 450 °C, respectively, followed by natural cooling to room temperature. After annealing, the absorption of the annealed photoanodes were enhanced, as confirmed by UV-vis-NIR measurements (Figure S2). However, the decrease of *J*_SC_ and *V*_OC_ was noticed especially after annealing at 350 °C, which eventually led to a decreased PCE (see Figure S3 for a detailed discussion of the effect of post-annealing). The failure of ZnS passivation and post-annealing in terms of further improving the performance of the CQDSCs indicates that the characteristics of this new type of solar cell are different from their counterparts based on ISQDs. This is understandable if one considers that the energy levels and/or charge transfer mechanisms behind CQDs and ISQDs are thought to be different [31]. Although the outcome of ZnS passivation and post-annealing is somehow disappointing, it indicates that further optimization of CQDSCs should adopt a method that is thoroughly different from that well-established for ISQDSCs, which is important in terms of fundamental research.

## 3. Materials and Methods

### 3.1. Materials

Citric acid and ethanediamine were obtained from Sinopharm Chemical Reagent Co. Ltd. Terpineol, ethyl cellulose (EC), P25, 200-nm-sized anatase TiO_2_ nanoparticles were purchased from Jingge Solar Co. Ltd. (Wuhan, China). All chemicals were used without further purification. Solutions for electrolyte and QD deposition were prepared with high-purity water obtained from a water purification system (Ulupure Instrument Co. Ltd. Chengdu, China). The electrode substrate was fluorine-doped tin oxide conducting glass (FTO, thickness: 2.2 mm, Nippon, sheet resistance 14 Ω/square).

### 3.2. Preparation of TiO_2_-Coated Photoanode

Two kinds of TiO_2_ pastes adopted for doctor blading were prepared using P25 and 200 nm TiO_2_ particles, respectively. A transparent TiO_2_ layer was first coated on FTO glass using P25 paste, which was further grown by a scattering layer with a 200 nm TiO_2_ paste. After being leveled for 15 min, the samples were heated at 80 °C for 30 min and then annealed at 450 °C for 30 min. Finally, the samples were treated in a 40 mM TiCl_4_ solution, rinsed with water, dried under air flow, and heated to 500 °C in air for 30 min.

### 3.3. In Situ Growth of CQDs on TiO_2_ Surface

A TiO_2_-coated photoanode is vertically immersed in the aqueous solution containing 0.5 mol·L^−1^ citric acid and 0.5 mol·L^−1^ ethanediamine. The system was then sealed in an autoclave at 180 °C and pyrolized for 24 h. The CQDs/TiO_2_ hybridized photoanode was rinsed with deionized water thoroughly and dried in air. The CdS/TiO_2_ hybridized photoanode was also prepared for comparison following the previous method (without annealing, photoanode 1 in Reference [1]).

For passivation with ZnS, the CQD-sensitized photoanode was dipped into 100 mmol·L^−1^ Zn(CH_3_COO)_2_ aqueous solution followed by dipping in Na_2_S aqueous solution with the same concentration. This dipping circle was repeated twice with a duration of 1 min. To see the post-annealing influence on the performance of the solar cell, the CQD-sensitized photoanode was also sintered at 350 °C and 450 °C, respectively, in a muffle furnace under nitrogen for 10 min.

### 3.4. Construction of the Solar Cell

To construct the solar cell, polysulfide electrolyte was sandwiched between the CQD- or CdS-sensitized photoanode and the counter electrode separated with a silicone spacer. The electrolyte contains 1 mol·L^−1^ Na_2_S and 1 mol·L^−1^·s in ethanol/water mixture (3/7, *v*/*v*). Cu_2_S on copper was selected as a counter electrode based on the literature. The active area of the solar cell is ~0.20 cm^2^.

### 3.5. Characterizations

SEM observations were carried out on JSM-6700F. TEM observations were performed on JEOL JEM-100 CXII (JEOL Co. Ltd., Tokyo, Japan). Samples were prepared by scraping the photoanodes from the FTO substrate and dispersed in ethanol solution, followed by transferring several drops of the suspension onto a carbon-coated copper grid. XRD patterns were obtained between 10° and 80° in the 2*θ* scan mode using a Rigaku D/Max 2200-PC diffractometer with Cu Ka radiation (*λ* = 0.15418 nm) and a graphite monochromator (Rigaku Co. Ltd., Tokyo, Japan) at room temperature. Photoluminescence was recorded on a PerkinElmer LS-55 fluorescence spectrometer. FTIR spectra were obtained on a Bruker ALPHA-T IR spectrophotometer. The absorbance spectra were recorded by U-4100 UV-vis-NIR spectrophotometer (Hitachi Co. Ltd., Tokyo, Japan).

The solar cell (active area: 0.20 cm^2^) was prepared in a sandwich structure and its performance was evaluated by a source meter (Keithley 2400) illuminated with a SS150A solar simulator (Zolix Instrument Co. Ltd., Beijing, China) under AM 1.5 illumination of 100 mW·cm^−2^. Electrochemical impedance spectra (EIS) were recorded in the sandwich cell using a CHI 600E electrochemical analyzer (Chenhua, Shanghai, China) in the dark. The measured frequency for EIS ranged from 100 kHz to 100 mHz, and the amplitude was set to 10 mV. The results were fitted by Zview software. The incident-photon-to-current conversion efficiency (IPCE) was measured by the DC method using an Solar Cell Scan 100 (IPCE system, Zolix Instrument Co. Ltd., Beijing, China) without bias illumination.

## 4. Conclusions

In summary, we have demonstrated a simple, low-cost, and effective strategy of growing CQDs in situ on TiO_2_ surfaces. The CQD-sensitized photoanode was then used to construct solar cell with a PCE of 0.87%, which is the highest record among CQDSCs. Compared to other CQDSCs reported previously, the solar cells demonstrated have higher PCEs and simplified fabrication method. Although the PCE is still much lower compared to that of the solar cells sensitized by ISQDs, the solar cells demonstrated here have a low cost and are environmentally friendly. By systematic optimization of photoanodes, counter electrode, and electrolyte, the performance of the CQDSCs could be further improved in future. Considering the fast growth in both fields related to solar cells and CQDs, there is no doubt that this new type of solar cell has a bright future.

## Supplementary Materials

The following are available online at http://www.mdpi.com/2079-4991/7/6/130/s1. Table S1: Key parameters of the solar cells sensitized by CQDs or GQDs reported in the literatures, Figure S1: *JV* curve of the CQDSC constructed with the photoanode passivated by ZnS, Figure S2: UV-vis-NIR absorption of CQD-sensitized photoanodes without and with post-annealing at different temperatures, Figure S3: *JV* characteristics of CQDSCs constructed by CQD-sensitized photoanodes without and with post-annealing at different temperatures.
