# Supplementary material for: Improving the Power Conversion Efficiency of Carbon Quantum Dot-Sensitized Solar Cells by Growing the Dots on a TiO2 Photoanode In Situ
Source: Nanomaterials (Basel). 2017 May 31;7(6):130. doi: 10.3390/nano7060130 (PMC5485777; doi:10.3390/nano7060130)
Supplement: Supplementary file 1 [file nanomaterials-07-00130-s001.pdf]

Electronic Supplementary Information

# Improving the Power Conversion Efficiency of Carbon Quantum Dot-Sensitized Solar Cells by Growing the Dots on a $TiO_2$ Photoanode In Situ

**Quanxin Zhang [1], Geping Zhang [2], Xiaofeng Sun [1], Keyang Yin [1] and Hongguang Li [1,*]**

[1] State Key Laboratory of Solid Lubrication & Laboratory of Clean Energy Chemistry and Materials, Lanzhou Institute of Chemical Physics, Chinese Academy of Sciences, Lanzhou 730000, China; qxzhang@licp.cas.cn (Q.Z.); sunxiaofengvip@licp.cas.cn (X.S.); yinky110403@163.com (K.Y.)

[2] Key Laboratory of Colloid and Interface Chemistry & Key Laboratory of Special Aggregated Materials, Shandong University, Ministry of Education, Jinan 250100, China; zgp0609@mail.sdu.edu.cn

* Correspondence: hgli@licp.cas.cn; Tel.: +86-931-496-8829

## 1. Summary of the previous reported solar cells

**Table S1.** Key parameters of the solar cells sensitized by CQDs or GQDs reported in the literatures.[a]

| Type of the dot | Precursors and preparation method | Photoanode | Hybridization for CQDs[b] | $J_{SC}$ (mA·cm$^{-2}$) | $V_{OC}$ (V) | *FF* | *PCE* (%) | Ref. |
|---|---|---|---|---|---|---|---|---|
| CQDs | γ-butyrolactone, acid reflux | $TiO_2$ | physical adsorption | 0.532 | 0.38 | 0.64 | 0.13 | 3 |
| | $CCl_4$ and $NaNH_2$, solvothermal | $TiO_2$ | hydrothermal[c] | 0.69 | 0.46 | 0.43 | 0.13 | 4 |
| | Chitosan-chitin, hydrothermal | ZnO (nanorods) | physical adsorption | 0.674 | 9.265 | 0.43 | 0.077 | 5 |
| | citric acid, urea, formic acid, microwave | $TiO_2$ | physical adsorption | 0.99 | 0.49 | 0.50 | 0.24 | 6 |
| | citric acid, ammonia, heating in the air | $TiO_2$ | physical adsorption (in acetone) | 2.65 | 0.47 | 0.625 | 0.79 | 7 |
| | citric acid, ethanediamine, hydrothermal | $TiO_2$ | *in situ* growth | 6.47 | 0.43 | 0.31 | 0.87 | This work |
| | citric acid, urea,microwave | $TiO_2$ | physical adsorption | only IPCE data were given | | | | 8 |
| | graphite rods, electrochemical | $TiO_2$ (nanotube) | physical adsorption (in acetone) | only EIS data were given | | | | 9 |
| GQDs | Multi-step organic synthesis | $TiO_2$ | physical adsorption (in toluene/ethanol) | 0.2 | 0.48 | 0.58 | ~0.06[d] | 10 |
| | Multi-step organic synthesis | $TiO_2$ | *in situ* synthesis | 2.49 | 0.55 | 0.64 | 0.87 | 11 |

[a] When multiple solar cells appear in the same literature, parameters of the best cell are extracted. [b] If not stated, the physical adsorption occurs in aqueous solutions. [c] Hydrothermal treatment between $TiO_2$-coated photoanode and pre-synthesized CQDs. [d] Calculated values based on $J_{SC}$, $V_{OC}$, FF and AM 1.5 Global light.

## 2. Additional data

2.1 Result from ZnS passivation

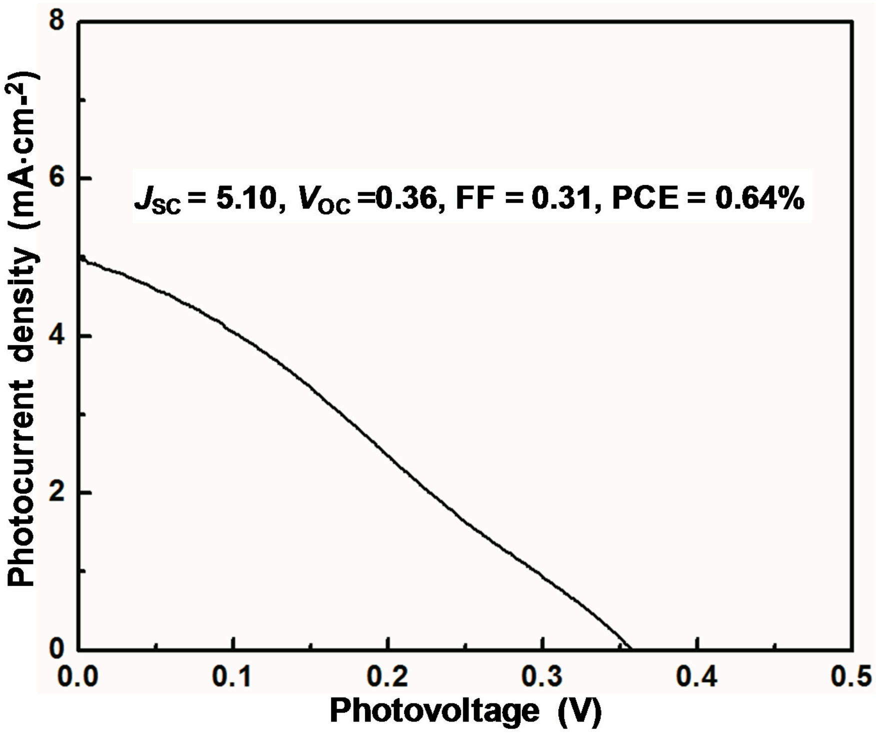


**Figure S1.** *JV* curve of the CQDSC constructed with the photoanode passivated by ZnS.

2.2 Results from post-annealing

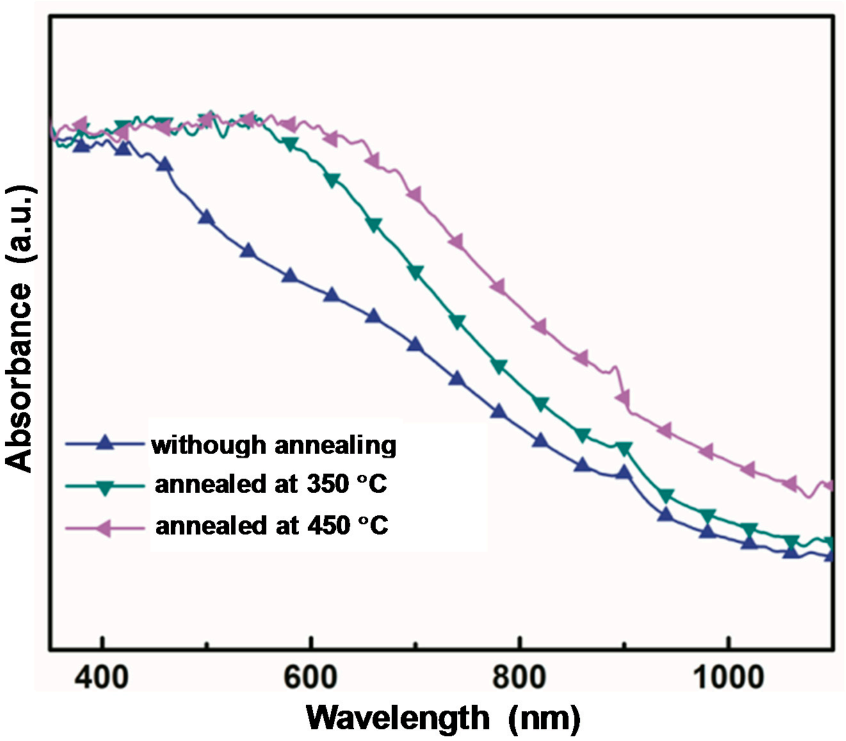


**Figure S2.** UV-vis-NIR absorption of CQDs-sensitized photoanodes without and with post-annealing at different temperatures.

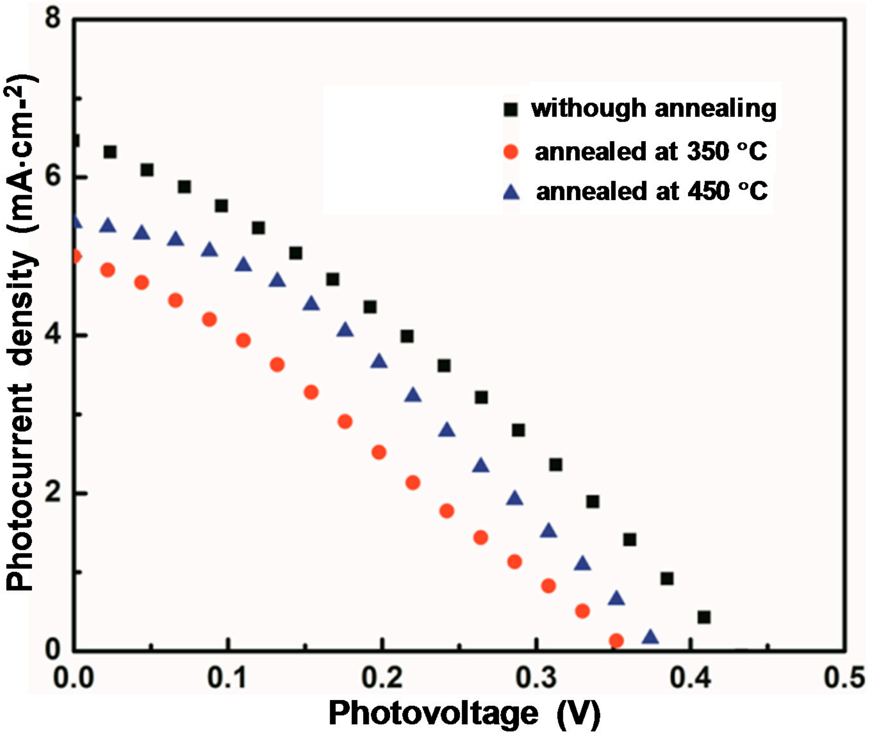


**Fig. S3** *JV* characteristics of CQDSCs constucted by CQDs-sensitized photoanodes without and with post-annealing at different temperatures.

We speculate that during annealing, at least four effects will be induced. i) The peripheral organic functional groups of the CQDs will be destroyed, which will induce a more compact contact between CQDs and $TiO_2$ and might facilitate charge transfer. ii) As the annealing was performed in nitrogen, the carbon produced by the burning of the organic functional groups will (at least partially) remain on the $TiO_2$ surfaces, accounting for the increase of absorbance of the annealed photoanodes. iii) The originally hydrophilic surfaces of CQDs will become hydrophobic after annealing, which will cause a nonideal contact between the photoanode and the electrolyte and increase the resistance between them. iv) Finally, the annealing will disturb the surface states of the CQDs which are believed to govern the PL characteristics of the CQDs. The influence of the last factor on the performance of the solar cell is currently unkown, as the detailed origin of the PL properties of CQDs is quite complicated and is still under debate. The obtained experimental results of the influence of annealing on the performance of the CQDSC could be the integrated results of the above-mentioned effects.

**References**


1. Q. Zhang, J. Cao and H. Li, *RSC Adv.*, 2015, **5**, 107957.

2. Q. X. Zhang, X. Z. Guo, X. M. Huang, S. Q. Huang, D. M. Li, Y. H. Luo, Q. Shen, T. Toyoda and Q. B. Meng, *Phys. Chem. Chem. Phys.*, 2011, **13**, 4659.

3. P. Mirtchev, E. J. Henderson, N. Soheilnia, C. M. Yip and G. A. Ozin, *J. Mater. Chem.*, 2012, **22**, 1265.

4. Y.-Q. Zhang, D.-K. Ma, Y.-G. Zhang, W. Chen, S.-M. Huang, *Nano Energy*, 2013, **2**, 545.

5. J. Briscoe, A. Marinovic, M. Sevilla, S. Dunn and M. Titirici, *Angew. Chem. Int. Ed.*, 2015, **54**, 4463.

6. J. T. Margraf, F. Lodermeyer, V. Strauss, P. Haines, J. Walter, W. Peukert, R. D. Costa, T. Clark and D. M. Guldi, *Nanoscale Horiz.*, 2016, **1**, 220.

7. H. Wang, P. Sun, S. Cong, J. Wu, L. Gao, Y. Wang, X. Dai, Q. Yi and G. Zou, *Nanoscale Res. Lett.*, 2016, **11**, 27.

8. M. Sun, S. Qu, W. Ji, P. Jing, D. Li, L. Qin, J. Cao, H. Zhang, J. Zhao and D. Shen, *Phys. Chem. Chem. Phys.*, 2015, **17**, 7966.

9. M. Sun, X. Ma, X. Chen, Y. Sun, X. Cui and Y. Lin, *RSC Adv.*, 2014, **4**, 1120.

10. X. Yan, X. Cui, B. Li and L. Li, *Nano Lett.*, 2010, **10**, 1869.

11. Z. Ji, R. Wu, L. Adamska, K. A. Velizhanin, S. K. Doorn and M. Sykora, *ACS Appl. Mater. Interfaces*, 2014, **6**, 20473.
